# Supplementary material for: Mapping mechanical stress in curved epithelia of designed size and shape
Source: Nat Commun. 2023 Jul 7;14:4014. doi: 10.1038/s41467-023-38879-7 (PMC10329037; doi:10.1038/s41467-023-38879-7)
Supplement: Supplementary file 3 — Reporting Summary [file 41467_2023_38879_MOESM3_ESM.pdf]

nature portfolio

Corresponding author(s): Xavier Trepat, Marino Arroyo, Sohan Kale

Last updated by author(s): May 10, 2023

Reporting Summary

Nature Portfolio wishes to improve the reproducibility of the work that we publish. This form provides structure for consistency and transparency in reporting. For further information on Nature Portfolio policies, see our Editorial Policies and the Editorial Policy Checklist.

Statistics

For all statistical analyses, confirm that the following items are present in the figure legend, table legend, main text, or Methods section.

n/a	Confirmed	
☐	☒	The exact sample size (*n*) for each experimental group/condition, given as a discrete number and unit of measurement
☐	☒	A statement on whether measurements were taken from distinct samples or whether the same sample was measured repeatedly
☐	☒	The statistical test(s) used AND whether they are one- or two-sided<br>*Only common tests should be described solely by name; describe more complex techniques in the Methods section.*
☐	☒	A description of all covariates tested
☒	☐	A description of any assumptions or corrections, such as tests of normality and adjustment for multiple comparisons
☐	☒	A full description of the statistical parameters including central tendency (e.g. means) or other basic estimates (e.g. regression coefficient) AND variation (e.g. standard deviation) or associated estimates of uncertainty (e.g. confidence intervals)
☐	☒	For null hypothesis testing, the test statistic (e.g. *F*, *t*, *r*) with confidence intervals, effect sizes, degrees of freedom and *P* value noted<br>*Give P values as exact values whenever suitable.*
☒	☐	For Bayesian analysis, information on the choice of priors and Markov chain Monte Carlo settings
☒	☐	For hierarchical and complex designs, identification of the appropriate level for tests and full reporting of outcomes
☒	☐	Estimates of effect sizes (e.g. Cohen's *d*, Pearson's *r*), indicating how they were calculated

Our web collection on statistics for biologists contains articles on many of the points above.

Software and code

Policy information about availability of computer code

Data collection	Micro-Manager (1.4.23) was used for imaging.
Data analysis	MATLAB version R2018b, MathWorks Inc., Natick, Massachusetts, 2018, and RStudio version 1.4.1717 (running R version 3.6.3) were used. Matlab was used for TFM calculation, cMSM implementation and image segmentation. RStudio was used for data analysis, statistical analysis and plotting.

For manuscripts utilizing custom algorithms or software that are central to the research but not yet described in published literature, software must be made available to editors and reviewers. We strongly encourage code deposition in a community repository (e.g. GitHub). See the Nature Portfolio guidelines for submitting code & software for further information.

Data

Policy information about availability of data

All manuscripts must include a data availability statement. This statement should provide the following information, where applicable:

- Accession codes, unique identifiers, or web links for publicly available datasets
- A description of any restrictions on data availability
- For clinical datasets or third party data, please ensure that the statement adheres to our policy

Source Data are provided with this paper

Research involving human participants, their data, or biological material

Policy information about studies with human participants or human data. See also policy information about sex, gender (identity/presentation), and sexual orientation and race, ethnicity and racism.

Reporting on sex and gender	N/A
Reporting on race, ethnicity, or other socially relevant groupings	N/A
Population characteristics	N/A
Recruitment	N/A
Ethics oversight	N/A

Note that full information on the approval of the study protocol must also be provided in the manuscript.

Field-specific reporting

Please select the one below that is the best fit for your research. If you are not sure, read the appropriate sections before making your selection.

☒ Life sciences ☐ Behavioural & social sciences ☐ Ecological, evolutionary & environmental sciences

For a reference copy of the document with all sections, see nature.com/documents/nr-reporting-summary-flat.pdf

Life sciences study design

All studies must disclose on these points even when the disclosure is negative.

Sample size	No statistical method was used to define sample size. For all experiments, a minimum of n=3 independent experiments were performed. Sample sizes were chosen based on practicality and throughput, and consistency with previously published similar works in the field.
Data exclusions	No data were excluded from the study.
Replication	Experiments were replicated at least thrice. Replication of the data was at all times successful. Experiments were performed on different days and with different cell batches, obtaining the same results.
Randomization	No randomization method was applied since data analysis was automatic or semi automatic
Blinding	Researchers were not blinded to group allocation during data collection and/or analysis. Blinding was not possible as the same researchers both produced and analysed the data. Data analysis was automatized to avoid possible biases.

Reporting for specific materials, systems and methods

We require information from authors about some types of materials, experimental systems and methods used in many studies. Here, indicate whether each material, system or method listed is relevant to your study. If you are not sure if a list item applies to your research, read the appropriate section before selecting a response.

Materials & experimental systems

n/a	Involved in the study	
☒	☐	Antibodies
☐	☒	Eukaryotic cell lines
☒	☐	Palaeontology and archaeology
☒	☐	Animals and other organisms
☒	☐	Clinical data
☒	☐	Dual use research of concern
☒	☐	Plants

Methods

n/a	Involved in the study	
☒	☐	ChIP-seq
☒	☐	Flow cytometry
☒	☐	MRI-based neuroimaging

Eukaryotic cell lines

Policy information about cell lines and Sex and Gender in Research

Cell line source(s)	All the experiments were carried out with a MDCK strain II line expressing CIBN–GFP–CAAX to visualize the plasma membrane. This cell line was created for the research published in:<br><br>doi: 10.1038/ncomms14396<br><br>The original cell line was provided by Dr Guillaume Charras (UCL)
Authentication	The cell line used was not authenticated.
Mycoplasma contamination	The cell line used in this work tested negative for Mycoplasma.
Commonly misidentified lines (See ICLAC register)	No commonly misidentified cell line was used in the study.
